# Engaging Patients and Professionals to Evaluate the Seriousness of Maternal and Child Health Outcomes: Protocol for a Modified Delphi Study

**DOI:** 10.2196/16478

**Published:** 2020-06-02

**Authors:** Lisa M Bodnar, Dmitry Khodyakov, Katherine P Himes, Jessica G Burke, Sara Parisi, Jennifer A Hutcheon

**Affiliations:** 1 Department of Epidemiology University of Pittsburgh Graduate School of Public Health Pittsburgh, PA United States; 2 Department of Obstetrics, Gynecology, and Reproductive Sciences University of Pittsburgh School of Medicine Pittsburgh, PA United States; 3 Magee-Womens Research Institute Pittsburgh, PA United States; 4 RAND Health Care Santa Monica, CA United States; 5 Department of Behavioral and Community Health Sciences University of Pittsburgh Graduate School of Public Health Pittsburgh, PA United States; 6 Department of Obstetrics and Gynaecology University of British Columbia Vancouver, BC Canada

**Keywords:** children, Delphi method, ExpertLens, mothers, pregnancy, patient engagement, online stakeholder engagement panels

## Abstract

**Background:**

Maternal weight gain during pregnancy is one of the few potentially modifiable risk factors for many adverse maternal and child health outcomes. Defining the optimal pregnancy weight gain range is difficult because, while lower weight gain may prevent some outcomes, such as maternal and child obesity, it may increase the risk of others such as fetal growth restriction and infant death. These health outcomes vary in their seriousness to mothers and their health care providers, and these differences in seriousness should be taken into account when determining optimal weight gain ranges. However, the relative seriousness that women and their care providers place on different health outcomes is unknown.

**Objective:**

We will determine the seriousness of 11 maternal and child health outcomes that have been consistently associated with pregnancy weight gain. We will achieve this by engaging patients and maternal and child health professionals using an online modified Delphi panel process.

**Methods:**

We aim to recruit a racially/ethnically and geographically diverse group of 90 US maternal and child health professionals and 90 women who are pregnant or less than 2 years postpartum. We will conduct 3 concurrent panels using the ExpertLens system, a previously evaluated online modified Delphi system that combines 2 rounds of rating with 1 round of feedback and moderated online discussion. In Round 1, panelists are asked to rate the seriousness of each health outcome on a scale of 0-100 and to provide a rationale for their scores. In Round 2, panelists will review their responses relative to those of other panelists. They will discuss their seriousness ratings anonymously using a moderated online discussion board. In Round 3, participants will revise their Round 1 responses based on group feedback and discussion. Each round will be open for 1-2 weeks.

**Results:**

The study protocol was reviewed by our ethics boards and did not require approval as human research. A pilot study of 6 professionals and 7 patients was completed in December 2019.

**Conclusions:**

Our numeric estimates of the seriousness of maternal and child health outcomes will enable future studies to determine pregnancy weight gain ranges that balance the risks of low and high weight gain for mothers and children.

**International Registered Report Identifier (IRRID):**

DERR1-10.2196/16478

## Introduction

Maternal and child health in the United States is far worse than expected from a high-income country. The US maternal mortality ratio (19 deaths per 100,000 live births) ranks 56th in the world, tied with Latvia, Romania, and Ukraine [1]. The infant mortality rate in the United States (6 deaths per 1000 live births) ranks 44th, behind Serbia, Poland, and Cuba [2]. These troubling statistics are driven in part by pregnancy complications (eg, preterm birth, gestational diabetes, preeclampsia, cesarean delivery, or a small-for-gestational-age infant) that occur in 1 of 3 US women [3]. Poor health at conception, including obesity and other chronic conditions, are also on the rise [3]. Despite decades of research, prevention of poor maternal and child health outcomes in the United States remains challenging.

Maternal weight gain during pregnancy is one of the few potentially modifiable risk factors for many maternal and child health outcomes [4]. Nevertheless, determining the range of pregnancy weight gain that optimizes maternal and child health is difficult. Although higher weight gain may reduce the likelihood of preterm birth, fetal growth restriction, and infant death, it may increase the risk of maternal obesity, gestational diabetes, preeclampsia, and childhood obesity [4-8]. Public health guidelines for pregnant women must identify the range of weight gain that minimizes the risks of both low and high weight gain for mothers and children.

In 2009, the Institute of Medicine (now the National Academy of Medicine) and National Research Council Committee to Reevaluate Gestational Weight Gain Guidelines sought to revise national weight gain recommendations such that they balanced maternal and infant risks associated with low and high pregnancy weight gain [4]. However, balancing risks is challenging because women and their care providers view some outcomes as more severe than other outcomes. For instance, a stillbirth is a more serious event than a cesarean delivery. Some complications, therefore, should carry more weight in the determination of optimal weight gain ranges.

The relative importance that women and their care providers place on different health outcomes is unknown. Although there are some tools for scoring adverse perinatal outcomes, they either do not consider outcomes for both the mother and child or do not include longer-term health outcomes [9-11]. Recognizing this limitation, the 2009 Institute of Medicine/National Research Council Committee commissioned a quantitative risk trade-off analysis [4]. Unfortunately, quantitative risk trade-off analysis requires estimates of health utility values, which quantify the preference, or value, that people place on a given health outcome, anchored in relation to death (a score of 0) and perfect health (a score of 1.0) [12]. However, health utility values have only been elicited for a limited number of adverse outcomes related to maternal-neonatal health [13]. As a result, the quantitative risk trade-off analysis was only able to account for the association between pregnancy weight gain and 3 health outcomes. With many other key outcomes left out, the relevance of the work was limited.

Our project was developed in response to the Institute of Medicine/National Research Council Committee’s call for research to fill this critical knowledge gap [4]. We aim to determine the importance of 11 maternal and child health outcomes that have been consistently associated with pregnancy weight gain. We will achieve this by engaging patients and maternal and child health professionals using an online modified Delphi panel process. The Delphi technique is a well-established method for exploring the existence of agreement among diverse stakeholder groups on a specific topic [14]. In an iterative process, panelists score items, provide a rationale for their ratings, review other panelists’ responses, and revise their initial scores. The process is anonymous, minimizing the negative effects of group decision making, such as groupthink.

Delphi panels have recently been used in perinatal research to achieve consensus on a list of core reporting outcomes for randomized trials of diet and lifestyle in pregnancy [15]. However, this study only sought to identify which components were important, and did not attempt to quantify their relative seriousness. A study by Oken et al [16] elicited weights on the seriousness of different health outcomes and incorporated these into a study associating pregnancy weight gain with adverse health outcomes; however, weights were only elicited for 5 health outcomes from a convenience sample of 12 Harvard researchers. Our project will build on this work by eliciting weights for a broad range of maternal and child health outcomes from a large, diverse, and multidisciplinary group of stakeholders, including both professionals and patients.

## Methods

### Participants

Although surveys aim to recruit a large, representative sample, the goal of an expert professional panel is to recruit the most knowledgeable individuals in the field to elicit their expertise.

#### Professionals

We will recruit 90 maternal and child health professionals in the United States who represent researchers, health care providers, public health experts, and policymakers from academic, government, or community sectors. Panel membership will therefore be chosen using content expertise as a primary selection criterion and ethnic/racial and geographic diversity as a secondary selection criterion.

We will recruit through our professional networks via email and social media. We will encourage respondents to nominate colleagues to participate. Interested participants will be asked to complete a study registration form, which will include questions about race/ethnicity, age, gender, state of residence, demographics, and professional background and experiences. We will use the responses to these questions to select participants. Participants must have access to the internet; they will be able to use any internet-connected device, including mobile phones. 

#### Patients

We will recruit 90 women who are pregnant or who carried a pregnancy to 20 weeks of gestation and are no more than 2 years postpartum. Women will not need to have had a poor outcome to be considered knowledgeable stakeholders. Our goal for participant recruitment is to ensure appropriate racial/ethnic and geographic variation. Purposeful sampling such as this is typical for stakeholder engagement panels [17].

We will recruit patients through social media. Interested patients will complete a screening form to determine eligibility. Participants must have access to the internet; they will be able to use any internet-connected device, including mobile phones. We will also inquire about age, race/ethnicity, state of residence, parity, whether they are currently pregnant or it has been less than 2 years since their last delivery, and how they heard about the study. We will use their responses to these questions to select participants.

### Panel Design

We will collect data using ExpertLens, an innovative online panel approach with a modified Delphi structure, created by researchers at the RAND Corporation [18,19]. This approach facilitates data collection, replaces traditional face-to-face meetings with anonymous moderated online discussion boards, and automates data collection. The ExpertLens platform can be accessed from any internet-connected device, including mobile devices. ExpertLens will allow the engagement of a large number of geographically diverse panelists by providing them with an opportunity to anonymously share their perspectives and interact with other participants using their own computer at their convenience [20]. The ExpertLens platform will save on costs and minimize the burden for participants that is typically associated with typical large national consensus meetings [20]. It has been used in numerous studies to elicit opinions from diverse stakeholder groups including researchers, providers, policymakers, patients, and community members [21-28].

We will conduct 3 concurrent panels using an identical research protocol. One panel will include 60 maternal and child health professionals. A second panel will include 60 pregnant or postpartum women. The final panel will include 30 professionals and 30 pregnant or postpartum women. Conducting homogeneous panels and a mixed panel will help explore the existence of consensus within and between the stakeholder groups and to explore the extent to which exposing participants in a mixed panel to alternative views could change their perspectives. A panel size of about 20-40 participants has been shown to create an engaging environment for online discussion. Our inclusion of 60 individuals per panel recognizes that participation rates in such panels vary from 50%-60% across the 3 rounds [29].

### Outcomes

We selected maternal and child health outcomes that will be rated on their importance by reviewing the 2009 Institute of Medicine/National Research Council scientific report [4], systematic reviews related to gestational weight gain, and other recent literature. We chose outcomes that have a consistent association with gestational weight gain in observational studies and can be clearly operationalized or measured in most research studies. We limited the health outcomes to no more than 12.

We developed background information for each outcome, including its definition and short- and long-term consequences [30-46]. We based this information primarily on UpToDate, a well-known evidence-based clinical resource. To make the information more accessible for patients, we relied on UpToDate’s The Basics text, which are short overviews written with plain language principles.

### Data Collection

Each panel will complete a 3-round ExpertLens process lasting approximately 4-5 weeks.

#### Round 1

**Figure 1:**
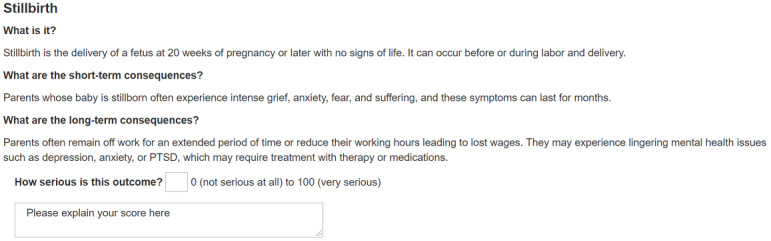
Mock-up of Round 1 graphic. PTSD: post-traumatic stress disorder.

In Round 1, we will ask panelists to review the background information provided for each outcome and rate each outcome on its importance (Figure 1). They will use a rating scale of 0-100, where 0 corresponds to not important at all and 100 corresponds to the most important. We chose this scale to mirror existing perinatal morbidity scoring tools, where severity points are assigned to adverse outcomes, and a score of 0 indicates a lack of morbidity [9]. In addition to scoring each outcome, panelists will be asked to provide rationales for their answers using open-text boxes.

#### Round 2

**Figure 2:**
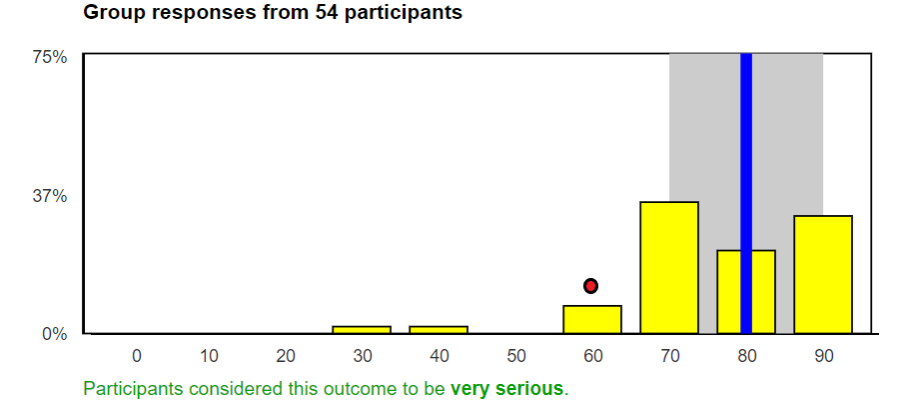
Mock-up of Round 2 graphic. The frequency of the 10 intervals of the entire panel’s ratings will be shown in yellow bars along with the group median (blue line), and the panelist’s own response (red dot).

In Round 2, panelists will see how their Round 1 responses compare to those of other panelists and review the group results (Figure 2). Participants will see the distribution of the entire panel’s ratings, their own ratings, and the ratings of each panelist who commented in Round 1 (listed with an anonymous identifier). For visual purposes, the panel’s ratings will be collapsed into 10 intervals (scores 0-9, 10-19, 20-29, etc) that will be displayed in a histogram. As there are 101 possible ratings (0-100, inclusive), the last interval will have 11 points and the remaining 9 intervals will have 10 points. The frequency of the 10 intervals of the entire panel’s ratings will be shown in yellow bars along with the group median (blue line), and the panelist’s own response (red dot). This statistical feedback is an important component of the Delphi process [47]. When the participant hovers over the chart, text boxes will appear to further assist in interpretation of the data. Further, instructional videos will assist in the interpretation of statistical results. In addition, participants will be notified if the panel was able to reach consensus on the importance of each complication. We will summarize the themes from the Round 1 open-ended comments in groups according to the rating (those who rated the outcome low, medium, or high seriousness). All panelists’ Round 1 comments will also be available for review.

In addition to reviewing a summary of all comments, participants will be able to respond to any other participant’s comment made in Round 1. Participants will discuss their ideas anonymously using an asynchronous moderated online discussion board [48]. The study team members, who are familiar with maternal and child health professionals and pregnant and postpartum women, have received training from RAND’s ExpertLens team members on how to serve as neutral moderators and will follow a recommended discussion moderator protocol [49] that included a moderator discussion and best practices manual. Moderators will promote active discussion, encourage participants to elaborate on responses, ask clarification questions, and ensure that a single participant does not dominate the discussion.

#### Round 3

In Round 3, participants will answer all Round 1 questions again based on Round 2 feedback and discussion. We will also ask panelists to provide their rationale for changing or maintaining their ratings for each outcome. Finally, panelists will be asked to complete a survey about their experience. We will ask open-ended questions about their participation experiences, factors that influenced their final seriousness ratings, and ways to improve the online engagement process. In addition, they will be asked to rate their satisfaction with the online engagement process using 7-point Likert-type scales. They will express their agreement with such statements as, “participation in this study was interesting,” “I was comfortable expressing my views in the discussion round,” and “the discussions brought out views I hadn’t considered.” These questions are intended to improve the ExpertLens process in subsequent panels.

All panelists will receive US $150 for completing all 3 rounds.

#### Pilot

The ExpertLens platform was pilot tested by one panel of 7 patients and one panel of 6 professionals. They participated in all three rounds of the ExpertLens process as if they were real study participants. Rounds 1 and 3 were open 1 day, and Round 2 was open 2 days. Moderators practiced generating discussion comments during Round 2 by posting a series of neutral questions and comments. After Round 3, each participant shared feedback on system usability, question clarity and readability, and the use of the discussion boards via a phone interview. Pilot participants received a US $150 gift card after the completion of all 3 rounds.

### Data Analysis

The primary analytic goal is to generate seriousness ratings for each maternal and child health outcome. Round 1 and 3 severity scores will be summarized for each panel by calculating the median, interquartile range (25th to 75th percentile), and maximum and minimum values. The severity scores that will be used in primary regression analyses to establish optimal gestational weight gain ranges in the next phase of our research will be the Round 3 median score for each outcome summed across the 3 panels. To establish the robustness of findings, we will perform sensitivity analyses by replacing the median Round 3 scores with (1) upper and lower values of the range of scores and (2) the median from each of the 3 panels. These analyses will allow the impact of any differences in scores on the optimal ranges to be quantified and incorporated into policymaking decisions.

Secondary analyses will include quantifying the degree of within-panel consensus of severity ratings using a validated process, the RAND/UCLA Appropriateness Method (RAM) [50]. ExpertLens uses this method to automatically determine the group decision (eg, whether a particular outcome was deemed serious by the panel) for each outcome. RAM quantifies the dispersion of scores in relation to the interpercentile range (30th to 70th percentiles). We will also examine differences in distributions between panels.

Thematic analysis will be used to explore the types of rating justifications and the impact of group dynamics. Qualitative data will include text responses to the open-ended questions in the discussion forums and on the surveys.

## Results

The ethics boards at University of Pittsburgh and The RAND Corporation determined the study protocol to be exempt from review. After completing the literature review to select the health outcomes of interest, we selected infant death, stillbirth, preterm birth, gestational diabetes, preeclampsia, small-for-gestational-age birth, large-for-gestational-age birth, unplanned cesarean delivery, obesity in women, childhood obesity, preterm birth, and metabolic syndrome in women.

We completed the pilot study and individual interviews with each of the 13 pilot testers. Based on their feedback, we made several key changes. First, we changed the wording of the panelists’ task from “rate the *importance* of each maternal and child health outcome” to “rate the *seriousness* of each maternal and child health outcome.” We operationalized seriousness as the severity of each condition based on the panelist’s overall impression of the outcome’s impact on a woman and child’s health and quality of life. Second, we changed the Round 1 instructions to include a list of all health outcomes that will be rated and factors the panelists should consider in their ratings: the short- or long-term nature of the outcome; how the outcome impacts quality of life; and consequences for those individuals close to the woman and the child (such as family, friends, or caregivers). We also noted that the ratings should not consider whether the outcome can be prevented; how common it is in the United States; or whether more research is needed to understand the outcome.

Third, we clarified the definition of a health outcome for the patient panel and the mixed composition panel by stating, “A ‘health outcome’ is a health condition, medical complication, diagnosis or negative event related to your health or your baby’s health.” Fourth, we standardized the background information for each health outcome using headings (definition, short-term complications, and long-term complications) and bulleted text below each heading with the intention of making this text more accessible for panelists with lower health literacy. We added consequences to quality of life in this information. Financial costs of each complication were removed from the background information because the available literature is not consistent in estimates, and how these relate to an average woman is difficult to determine given variation in insurance coverage.

In the pilot study, we found that there was less discussion for the outcomes that appeared at the end of the list. We modified the ExpertLens system to randomize the outcomes for each panelist. The study was completed in December 2019.

## Discussion

Our work will support the development of evidence-based pregnancy weight gain recommendations. The seriousness ratings for each individual outcome that our study generates will be used to develop a severity-weighted composite outcome that we will study in relation to gestational weight gain. This analysis will allow us to quantitatively account for expert and stakeholder opinion on the seriousness of these health outcomes. This will permit a determination of the range of pregnancy weight gain at which risks of adverse outcomes for mothers and children are balanced. Our incorporation of the perspectives of currently or recently pregnant women in this work is novel and important because it will help make the results of this work more patient-centered and will highlight any differences between the perspectives of experts and patients. As such, our project is consistent with the growing trend toward engaging patients in the development of evidence-based clinical practice guidelines. Our incorporation of the perspectives of current or recently pregnant women in this work is novel and important because it will help make the results of this work more patient-centered and will highlight any differences between the perspectives of experts and patients. As such, our project is consistent with the growing trend toward engaging patients in the development of evidence-based clinical practice guidelines [51,52].

We recognize that quantifying the perceived severity of different health outcomes is challenging, and panelists may not come to a consensus on the outcomes’ relative seriousness. If there is no consensus, we believe that this represents the reality of the diverse experiences of women and care providers and should be reported in the literature. By reporting not only median scores, but also the range of scores for a given outcome and exploring the extent to which each panel was able to reach consensus, our work will enable researchers to explore the impact of different weights through sensitivity analyses that use the highest and lowest values elicited from each panel. These findings will also inform policymakers on the magnitude of variation in optimal ranges obtained from diverse opinions and account for the complex trade-off between low and high weight gain on maternal and child health.

Additionally, our project will illustrate a methodology for incorporating stakeholder perspectives on optimal treatment exposure beyond gestational weight gain. This may be especially important for treatments that are linked with multiple, competing adverse health outcomes that differ in their seriousness to patients and providers. For example, such a methodology could be used to aid decision making for establishing optimal birth spacing (balancing risks of long spacing due to preeclampsia and infertility with risks of short spacing due to preterm birth) [53]. Other controversial areas where this methodology may be employed are decision making regarding antidepressant use during pregnancy [54] and vaginal birth after cesarean delivery [55]. Quantifying the seriousness of different health outcomes is a critical first step toward ensuring that optimal public health recommendations are both evidence-based and reflect the values of women and their care providers.

## Appendix

Multimedia Appendix 1 Peer review of funded grant proposal.

## Abbreviations

RAM: RAND/UCLA Appropriateness Method
