# Supplementary material for: Engaging Patients and Professionals to Evaluate the Seriousness of Maternal and Child Health Outcomes: Protocol for a Modified Delphi Study
Source: JMIR Res Protoc. 2020 Jun 2;9(6):e16478. doi: 10.2196/16478 (PMC7298634; doi:10.2196/16478)
Supplement: Multimedia Appendix 1 [file resprot_v9i6e16478_app1.pdf]

SUMMARY STATEMENT

(Privileged Communication)

PROGRAM CONTACT:
Menachem Miodovnik
301-451-5031
menachem.miodovnik@nih.gov

Release Date: 07/19/2018
Revised Date:

Application Number: 1 R01 HD094777-01A1

Principal Investigators (Listed Alphabetically):

BODNAR, LISA M (Contact)
HUTCHEON, JENNIFER ANNE

Applicant Organization: UNIVERSITY OF PITTSBURGH AT PITTSBURGH

Review Group: IRAP
Infectious Diseases, Reproductive Health, Asthma and Pulmonary Conditions Study Section

Meeting Date: 06/26/2018
Council: OCT 2018
Requested Start: 12/01/2018

RFA/PA: PA18-484
PCC: PPB -ZZ
Dual PCC: RAJ DUAL
Dual IC(s): DK

Project Title: Innovative approaches to inform evidence-based pregnancy weight gain guidelines

SRG Action: Impact Score:15 Percentile:1
Next Steps: Visit https://grants.nih.gov/grants/next_steps.htm
Human Subjects: 30-Human subjects involved - Certified, no SRG concerns
Animal Subjects: 10-No live vertebrate animals involved for competing appl.
Gender: 2A-Only women, scientifically acceptable
Minority: 1A-Minorities and non-minorities, scientifically acceptable
Children: 1A-Both Children and Adults, scientifically acceptable

Project Year	Direct Costs Requested	Estimated Total Cost
1	496,150	686,242
2	499,998	691,564
3	438,372	606,327
4	450,472	623,063
TOTAL	1,884,992	2,607,195

ADMINISTRATIVE BUDGET NOTE: The budget shown is the requested budget and has not been adjusted to reflect any recommendations made by reviewers. If an award is planned, the costs will be calculated by Institute grants management staff based on the recommendations outlined below in the COMMITTEE BUDGET RECOMMENDATIONS section.

1R01HD094777-01A1 Bodnar, Lisa

RESUME AND SUMMARY OF DISCUSSION: In this application, the investigators propose to better characterize the relationship between gestational weight gain and maternal child health outcomes to improve current maternal weight gain guidelines. The reviewers agreed that this application addresses significant knowledge gaps that compromise the value of current Institute of Medicine pregnancy guidelines surrounding the modifiable risk factor of gestational weight gain. A particular area of significance for this application is addressing lack of research on pregnancy weight gain in obese and severely obese women. A great innovation of this application discussed is the consideration of the severity of the maternal fetal health outcomes with the novel application of the Delphi-consensus process to obstetrics care. This resubmission was found greatly responsive previous critique with changes like making the study cohort more contemporary and generalizable incorporating greater racial and ethnic diversity. The sophisticated analytical plan also demonstrates the refinement of the study processes including the proposed prospective study of weight gain prior and following clinical outcomes. Following discussion, the committee concluded that this application is likely to have a high impact to provide the scientific basis to improve upon current pregnancy weight gain guidelines.

DESCRIPTION (provided by applicant): A large volume of research has shown that excessive pregnancy weight gain contributes to maternal postpartum weight retention, child obesity, cesarean delivery, gestational diabetes and preeclampsia, while low gestational weight gain is linked with fetal growth restriction, preterm birth, and perinatal death. However, major gaps remain in our ability to convert these findings into evidence-based recommendations on optimal pregnancy weight gain. The status quo is to evaluate each outcome separately, and without regard to its perceived severity. Further, subjective approaches have been used to select the cut-points of recommended weight gain ranges. Policy-makers urgently need evidence on the optimal range of pregnancy weight gain that considers multiple adverse outcomes simultaneously, accounts for differences in the relative severity of different outcomes, and establishes cut-points using systematic and reproducible methods. In this application, we will develop and apply advanced methodologies that support the creation of robust, evidence-based public health recommendations for weight gain in pregnancy. First, we will generate weights reflecting stakeholder perspectives on the relative severity of competing maternal and child health outcomes associated with gestational weight gain. A Delphi-consensus process will summarize the perceived severity of competing maternal and child health outcomes from diverse panels of 60 women and 60 content experts. Second, we will use a large, diverse cohort of U.S. pregnant women to determine the relationship between gestational weight gain and a composite outcome of adverse events for mothers (preeclampsia, gestational diabetes, cesarean delivery, longer-term obesity, subclinical cardiovascular disease) and children (perinatal death, preterm birth, and small- and large-for-gestational-age birth), with each component weighted to account for its relative severity. Then, we will use our previously-published quantitative approach to identify the optimal range of pregnancy weight gain for women according to different body mass index values, defined as the weight gain associated with the lowest risks of combined, severity-weighted maternal and child outcomes. Successful completion of these aims will provide policy makers with evidence on the complex trade-offs between low and high weight gain on short- and longer-term maternal and child health in order to inform pregnancy weight gain guidelines. Our innovative approach to study multiple adverse outcomes simultaneously and account for their relative severity will enable us to overcome challenges that have previously impeded the synthesis of existing research studies into a systematic, evidence-based weight gain guideline. Our work will impact public health and medical practice by providing high quality evidence for optimizing the nutritional care of pregnant mothers and children.

PUBLIC HEALTH RELEVANCE: The 2009 Institute of Medicine pregnancy weight gain guidelines are used by clinicians throughout the U.S. to optimize the short- and long-term health of both the mother and child, yet there are still major gaps in knowledge about ideal weight gain ranges. This project will provide data to inform future evidence-based weight gain guidelines by using cutting-edge analytic

methodologies to synthesize individual associations between weight gain and multiple adverse health outcomes of major medical, economic, and public health importance, including perinatal death, poor birth outcomes, and obesity. Although the causes of poor maternal and offspring outcomes are multifactorial, we focus on pregnancy weight gain because the association is plausible, potentially modifiable, and has important implications for mother and child well beyond the childbearing years.

CRITIQUE 1

Significance: 2
Investigator(s): 1
Innovation: 1
Approach: 2
Environment: 1

Overall Impact: This is a resubmission of an Multiple Principal Investigator application from Bodnar and Hutcheon. This project is focused on establishing scientifically justified maternal weight gain guidelines that balance competing maternal and fetal risks, and explicitly consider the severity of the outcomes under study. The Principal Investigators have been responsive to prior reviews by changing the cohort to one that is generalizable to the US population, and by providing a more detailed justification for the Delphi method of consensus building. This is a significant area of research because 1/3 US women are obese, excessive weight gain during pregnancy is modifiable and is also a driver of the obesity epidemic, and current guidelines were chosen by the IOM in an ad hoc manner, and had insufficient evidence in several areas including: the inability to separate weight gain before and after outcomes like preeclampsia and gestational diabetes (concerns about reverse causality), and weight gain recommendations for severely obese women (a growing population of concern). This research will therefore directly inform clinical guidelines and help to identify populations in need of intervention. Score driving issues include the large, diverse, US population-based cohort with a substantial fraction of women in the obese and severely obese categories (19% BMI >=30); prospective weight measurements allowing the separation of weight gain prior to or following diagnosis; consideration of outcome severity; and excellent investigator team with long record of collaborative work in this area. If successful, this research will have a high impact on the field, particularly for under-researched populations such as the obese and severely obese women for whom evidenced-based guidelines are thin.

1. Significance:

Strengths

- This is a significant area of research because 1/3 US women are obese, excessive weight gain during pregnancy is modifiable and is also a driver of the obesity epidemic
- Current guidelines were chosen by the IOM in an ad hoc manner, and had insufficient evidence in several areas including: the inability to separate weight gain before and after outcomes like preeclampsia and gestational diabetes (concerns about reverse causality), and weight gain recommendations for severely obese women (a growing population of concern).
- The most significant contribution of this study is better evidence-based guidelines for the obese and severely obese women, a growing segment of the population.
- Additionally, prior guidelines considered a fairly narrow range of competing outcomes given a dearth of information and long-term follow-up. This study is positioned to consider more outcomes and additionally will explicitly balance competing maternal and fetal priorities.

Weaknesses

- It's not clear how worrisome a cohort of only nulliparous women is. In some ways it could be good in that a higher proportion would be at risk for outcomes like preeclampsia. However, weight gain may be variable by parity, and this cohort will not be able to address parity-related issues. If parity is a confounder, then a nulliparous cohort is fine. If parity is an effect modifier, we will not be able to observe differences. Overall I judge this to be a minor weakness and the benefits of the data source outweigh this limitation.

2. Investigator(s):

Strengths

- This investigator team has a long record of collaboration in this area.
- Dr. Bodnar was a member of the original IOM panel that established the current weight gain guidelines, and is an expert in the area of weight gain in pregnancy.
- Dr. Hutcheon is a statistically oriented perinatal epidemiologist with strong record of productivity
- The investigator team is excellent

Weaknesses

- None noted

3. Innovation:

Strengths

- Use of Delphi method to establish weights for severity
- Breadth of outcomes considered
- Cohort with prospective weights

Weaknesses

- None noted

4. Approach:

Strengths

- Delphi panel approach is innovative and well defended. Online format is ideal given that it will allow for a geographically diverse panel at minimal cost, and will minimize negative effects of group decision making.
- LOS provided by several panelists.
- Decision making process very clearly described.
- nuMoM2b replaces old cohorts, Includes about 10K nulliparous women, with adequate racial/ethnic diversity and a substantial fraction of obese/severely obese women (19%).
- Availability of prospective weight measurements will help to guard against reverse causality for outcomes that occur during pregnancy (e.g. preeclampsia and gestational diabetes).
- 90% follow-up of women 2-7 years postpartum.
- Availability of important postpartum outcomes such as excess maternal postpartum weight retention and maternal postpartum metabolic syndrome.
- Timeline adequate for aims and scope of work

Weaknesses

- I have some concerns about weighting guidelines only by severity and not prevalence. While I agree with the investigators that perinatal death is more important than c-section (for example), the prevalence of one is orders of magnitude higher than the other. Shouldn't severity be balanced by prevalence?
- Some concern about cohort only being nulliparas. Overall I think this is minor concern when balanced against strength of the resource.

5. Environment:

Strengths

- Excellent for proposed research

Weaknesses

- None noted

Study Timeline

Strengths

Weaknesses

- None noted

Protections for Human Subjects:

Acceptable Risks and/or Adequate Protections

Data and Safety Monitoring Plan (Applicable for Clinical Trials Only):

Not Applicable (No Clinical Trials)

Inclusion of Women, Minorities and Children:

- Sex/Gender: Distribution justified scientifically
- Race/Ethnicity: Distribution justified scientifically
- For NIH-Defined Phase III trials, Plans for valid design and analysis: Not applicable
- Inclusion/Exclusion of Children under 18: Including ages <18; justified scientifically

Vertebrate Animals:

Not Applicable

Biohazards:

Not Applicable

Resubmission:

- The investigators have been very responsive to prior concerns. The major changes include dropping the previous cohorts and switching to the nuMoM2b cohort, which includes 10,000 nulliparas from across the US. This cohort is also pretty contempoaray (2010-2013). This

enhances the generalizability of the results. The investigators also provide a stronger rationale for the application of the Delphi consensus approach for balancing maternal and fetal risks.

Resource Sharing Plans:

Not Applicable

Authentication of Key Biological and/or Chemical Resources:

Not Applicable

Budget and Period of Support:

Recommend as Requested

CRITIQUE 2

Significance: 2
Investigator(s): 1
Innovation: 2
Approach: 2
Environment: 1

Overall Impact: In this project, the investigators propose a method to produce new and better-informed guidelines for gestational weight gain compared the IOM guidelines. They propose to use a modified Delphi approach to develop weights for pregnancy and child adverse outcomes based on importance of the outcomes to both an expert panel and a consumer group made up of pregnant women or moms. They will then use these weights along with a Poisson regression to identify optimal gestational weight gain to avoid adverse outcomes. They will perform sensitivity analyses to verify that the guidelines obtained are indeed the optimal values for gestational weight gain.

1. Significance:

Strengths

- The current IOM guidelines for optimal weight gain in pregnancy are somewhat lacking in their origins. This project aims to utilize the nuMoM2b dataset to refine the guidelines for weight gain during pregnancy to improve both maternal and child outcomes.

Weaknesses

- While the current guidelines are not ideal, they do provide guidance which is not often adhered too. It is unclear as to whether guidelines provided by the current project would have any better adherence. Although the investigators have no influence on this, it does temper the significance slightly.

2. Investigator(s):

Strengths

- Highly qualified team with little overlap in expertise so very little duplication will occur.

- The Multiple Principal Investigators have complementary expertise and have a successful track record of working together.

Weaknesses

- None noted

3. Innovation:

Strengths

- The use of the modified Delphi process in establishing weights for adverse pregnancy outcomes is unique to this type of study.

Weaknesses

- The work being proposed is a logical step forward from work already completed by the investigators.

4. Approach:

Strengths

- Composition of the scoring groups is well considered and while there may be more participation in the expert group, the investigators have planned well enough to make participation easy as possible for the 'consumer' group.
- Good use of Poisson modeling to address the complications of the distribution of the data.
- Appears to be appropriate power to evaluate weight gain by quintile (not BMI group) and compare the extreme quintiles with the middle quintile.
- Good use of pre-diagnosis of GDM and preeclampsia weight gain which is then compared to the weight gain in the rest of the sample to impute a final weight gain value.
- Excellent plan to do sensitivity analysis to determine the impact of the difference in scoring by the two groups.

Weaknesses

- The only consideration of biological variables is within the covariates included in the model – race/ethnicity, fetal sex, maternal age. It is possible that to really consider these variables subgroup analysis, for which there might be very little power, would need to be conducted at least on an exploratory level to determine if further investigation is warranted.
- Current weight gain guidelines are different based on pregravid weight category. It is unclear if the current project would provide a single weight gain goal or if it would also be able to provide the goals by BMI group. The IOM also provides weight gain guidance for 2nd and 3rd trimester which seems totally lacking in this project. While this was probably intended it is not explicitly stated in the application.
- Simulation studies could be performed to give a better guide to power/sample size for this model, although given the sample size available the investigators are likely to continue to be able to determine risk at similar levels.
- Since enrollment is in the first trimester, would it be better to use enrollment weight rather than self-reported pre-pregnancy weight? Self-reported weight is habitually flawed and first trimester weight would be fairly similar to pre-pregnancy weight.

- Proposal says there will be adjustment for gravidity but all women are nulliparous in nuMoM2b so there is no need for adjustment.

5. Environment:

Strengths

- The environment is outstanding at the various sites.

Weaknesses

- None noted

Protections for Human Subjects:

Acceptable Risks and/or Adequate Protections

- No new collection of data and all data transferred from the nuMoM2b coordinating center will be deidentified.

Data and Safety Monitoring Plan (Applicable for Clinical Trials Only):

Not Applicable (No Clinical Trials)

Inclusion of Women, Minorities and Children:

- Sex/Gender: Distribution justified scientifically
- Race/Ethnicity: Distribution justified scientifically
- For NIH-Defined Phase III trials, Plans for valid design and analysis:
- Inclusion/Exclusion of Children under 18: Including ages <18; justified scientifically
- Only women included as we are dealing with pregnancy. Will include all moms from the nuMoM2b which covers ages 13-45 and a mixture of race/ethnicities. No one is excluded based on age or race/ethnicity in this study.

Vertebrate Animals:

Not Applicable (No Vertebrate Animals)

Biohazards:

Not Applicable (No Biohazards)

Resubmission:

- The investigators did a nice job in addressing the major issues from the initial review. They adjusted the data being used to no longer use a dataset from Sweden which would have limited the generalization to the at large US population.

Select Agents:

Not Applicable (No Select Agents)

Authentication of Key Biological and/or Chemical Resources:

Not Applicable (No Relevant Resources)

Budget and Period of Support:

Recommend as Requested

CRITIQUE 3

Significance: 1
Investigator(s): 1
Innovation: 1
Approach: 2
Environment: 1

Overall Impact: This group of established investigators propose to fill knowledge gaps in understanding the relationship between gestational weight gain and maternal and child health outcomes by applying the Delphi method, an approach which is relatively novel for obstetrical healthcare. The overarching aim is to better inform healthcare providers and policy makers in defining weight gain thresholds across pregnancy by focusing on both short- and long-term health outcomes of both mothers and babies.

The researchers have been thoughtful in their response to the previous critique of this application which in essence focused on the use of a Swedish birth cohort for comparison and potential compromise in reproducibility, lack of clarity with regards to the total number of stakeholders for the interviews and timeline for study conduct. With this resubmission, the researchers instead now will use the contemporary cohort of nearly 10,000 pregnancies from the US nuMoM2b cohort. These are mothers who were recruited from 8 academic sites across the nation as a longitudinal cohort to evaluate various features which factor into a variety of different adverse perinatal outcomes. Maternal and neonatal outcomes are exquisitely characterized. Added information explaining and justifying the number of stakeholders to be interviews and a reduction in the time required to conduct the investigation are also features of this resubmission.

This new approach is novel and should advance the field by informing future gestational weight gain guidelines; the work leverages the expertise of the researchers who were contributors to the 2009 Institutes of Medicine Guidelines, including Principal Investigator, Lisa Bodnar, PhD, on gestational weight gain in pregnancy and reflects the coalescence of leaders in the field. The impact is deemed to be high.

1.Significance:

Strengths

- This application addresses a significant knowledge gap in the thresholds for gestational weight gain (GWG) and should move the field forward immensely.
- The scientific premise is sound insofar as inadequate and excessive GWG have both been identified as being associated with adverse maternal and neonatal short- and long-term outcomes.
- The predicate for the extant IOM thresholds are often based on somewhat specious data.

Weaknesses

- None noted

2. Investigator(s):

Strengths

- Dr. Bodnar has amassed an exceptionally well-qualified and well-honed team for this work. She herself was a member of the IOM committee for the 2009 report and brings her expertise in maternal nutrition. She is also well-versed in policy and advocacy and will be anticipated to lend that expertise to the results of this investigation as well; tremendous impact is anticipated.
- Dr. Hutcheon is a perinatal epidemiologist with whom Dr. Bodnar has worked and published previously.
- Other members include Barbara Abrams to lend her expertise in GWG and (NAME) Himes as a clinical maternal-fetal medicine specialist. Robert Platt from McGill University will serve as biostatistician and Ann Yaktine, Director of the Food and Nutrition Board of the National Academies of Sciences will also serve as a consultant.
- Dr. Dmitry Khordyakov is a Behavioral/Social Scientist at RAND Health who is a co-developer of ExpertLens, the primary tool to be used for this investigation.

Weaknesses

- None noted

3. Innovation:

Strengths

- Application of the Delphi method with 60 interviewees from various stakeholder parties is innovative and exciting. This will be used to support development of an outcome severity index based on GWG strata.
- Novel approach employed for identifying must appropriate cut-points for weight-gain guidelines developed from non-inferiority approaches used in clinical trials which have been carefully cited in this application.

Weaknesses

- None noted

4. Approach:

Strengths

- See above re: application of Delphi method with input from researchers from the Rand corporation, leveraging existing data from clinical trials in an attempt to redefine approach thresholds for GWG.
- Use of two large delivery databases: a cohort of over 140,000 deliveries from Magee-Women's Hospital in Pittsburgh that has been developed and was used for prior work focused on analysis of each of 11 outcomes individually; and modification of resubmitted application to use NuMoM2b data from a contemporary American birth cohort of over 10,000 births. This change in methodology vastly improves relevance and generalizability.
- Large sample sizes lend themselves to adequate power.
- Timeline has been revised reflecting a reduction by one year.
- Justification for the two panels of 60 stakes holders (including women/patients/patient advocates as well as healthcare professionals) included in response to previous critique.

Weaknesses

- If there is a need to identify any weakness in this application, the considerations re: race/ethnicity seem less robust than one might otherwise expect. That said, knowing the experience and expertise of this team, there is no doubt in this reviewer's mind that the effect of race/ethnicity on GWG will be considered in multiple ways.

5. Environment:

Strengths

- Outstanding work environment overall.

Weaknesses

- None noted

Protections for Human Subjects:

Acceptable Risks and/or Adequate Protections

Data and Safety Monitoring Plan (Applicable for Clinical Trials Only):

Not Applicable (No Clinical Trials)

Inclusion of Women, Minorities and Children:

- Sex/Gender: Distribution justified scientifically
- Race/Ethnicity: Distribution justified scientifically
- For NIH-Defined Phase III trials, Plans for valid design and analysis: Not applicable
- Inclusion/Exclusion of Children under 18: Including ages <18; justified scientifically

Vertebrate Animals:

Not Applicable (No Vertebrate Animals)

Biohazards:

Not Applicable (No Biohazards)

Applications from Foreign Organizations:

Not Applicable (No Foreign Organizations)

Select Agents:

Not Applicable (No Select Agents)

Resource Sharing Plans:

Acceptable

Authentication of Key Biological and/or Chemical Resources:

Not Applicable (No Relevant Resources)

Budget and Period of Support:
Recommend as Requested

THE FOLLOWING SECTIONS WERE PREPARED BY THE SCIENTIFIC REVIEW OFFICER TO SUMMARIZE THE OUTCOME OF DISCUSSIONS OF THE REVIEW COMMITTEE, OR REVIEWERS' WRITTEN CRITIQUES, ON THE FOLLOWING ISSUES:

PROTECTION OF HUMAN SUBJECTS: ACCEPTABLE

INCLUSION OF WOMEN PLAN: ACCEPTABLE

INCLUSION OF MINORITIES PLAN: ACCEPTABLE

INCLUSION OF CHILDREN PLAN: ACCEPTABLE

COMMITTEE BUDGET RECOMMENDATIONS: The budget was recommended as requested.

Footnotes for 1 R01 HD094777-01A1; PI Name: Bodnar, Lisa M

NIH has modified its policy regarding the receipt of resubmissions (amended applications). See Guide Notice NOT-OD-14-074 at http://grants.nih.gov/grants/guide/notice-files/NOT-OD-14-074.html. The impact/priority score is calculated after discussion of an application by averaging the overall scores (1-9) given by all voting reviewers on the committee and multiplying by 10. The criterion scores are submitted prior to the meeting by the individual reviewers assigned to an application, and are not discussed specifically at the review meeting or calculated into the overall impact score. Some applications also receive a percentile ranking. For details on the review process, see http://grants.nih.gov/grants/peer_review_process.htm#scoring.

MEETING ROSTER

Infectious Diseases, Reproductive Health, Asthma and Pulmonary Conditions Study Section
Population Sciences and Epidemiology Integrated Review Group
CENTER FOR SCIENTIFIC REVIEW
IRAP
06/26/2018 - 06/27/2018

Notice of NIH Policy to All Applicants: Meeting rosters are provided for information purposes only. Applicant investigators and institutional officials must not communicate directly with study section members about an application before or after the review. Failure to observe this policy will create a serious breach of integrity in the peer review process, and may lead to actions outlined in NOT-OD-14-073 at https://grants.nih.gov/grants/guide/notice-files/NOT-OD-14-073.html and NOT-OD-15-106 at https://grants.nih.gov/grants/guide/notice-files/NOT-OD-15-106.html, including removal of the application from immediate review.

CHAIRPERSON(S)

MORRIS, JOHN GLENN JR, MD, MPH
PROFESSOR
EMERGING PATHOGENS INSTITUTE
SCHOOL OF MEDICINE
UNIVERSITY OF FLORIDA
GAINESVILLE, FL 32611

MEMBERS

ALLARD, PATRICK, PHD *
ASSISTANT PROFESSOR
DEPARTMENT OF ENVIRONMENTAL HEALTH SCIENCES
FIELDING SCHOOL OF PUBLIC HEALTH
UNIVERSITY OF CALIFORNIA, LOS ANGELES
LOS ANGELES, CA 90095

BANCALARI, EDUARDO H, MD *
PROFESSOR OF PEDIATRICS, OBSTETRICS AND GYNECOLOGY
DEPARTMENT OF PEDIATRICS
DIVISION OF NEONATOLOGY
UNIVERSITY OF MIAMI/JACKSON MEMORIAL MED CTR
MIAMI, FL 33101

BARNHART, KURT T, MD, MSCE
PROFESSOR
PENN FERTILITY CARE
UNIVERSITY OF PENNSYLVANIA
PHILADELPHIA, PA 19104

BRETON, CARRIE VAN DOREN, SCD *
ASSOCIATE PROFESSOR
DEPARTMENT OF PREVENTIVE MEDICINE
KECK SCHOOL OF MEDICINE
UNIVERSITY OF SOUTHERN CALIFORNIA
LOS ANGELES, CA 90089

CHRISTOFFERSON, REBECCA CARRIERE, PHD *
ASSISTANT PROFESSOR
DEPARTMENT OF PATHOBIOLOGICAL SCIENCES
LOUISIANA STATE UNIVERSITY
BATON ROUGE, LA 70803

COULL, BRENT, PHD
PROFESSOR
DEPARTMENT OF BIOSTATISTICS
T.H. CHAN SCHOOL OF PUBLIC HEALTH
HARVARD UNIVERSITY
BOSTON, MA 02115

DARROW, LYNDSEY, PHD *
ASSOCIATE PROFESSOR
SCHOOL OF COMMUNITY HEALTH SCIENCES
UNIVERSITY OF NEVADA, RENO
RENO, NV 89557

DEL VALLE, SARA, PHD
DEPUTY GROUP LEADER
ANALYTICS, INTELLIGENCE AND TECHNOLOGY DIVISION
LOS ALAMOS NATIONAL LABORATORY
LOS ALAMOS, NM 87545

DREWS, KIMBERLY LEE, PHD *
ASSOCIATE RESEARCH PROFESSOR
DEPARTMENT OF EPIDEMIOLOGY AND BIOSTATISTICS
SCHOOL OF PUBLIC HEALTH AND HEALTH SERVICES
THE GEORGE WASHINGTON UNIVERSITY
ROCKVILLE, MD 20852

ENGEL, STEPHANIE M, MSPH, PHD
PROFESSOR
DEPARTMENT OF EPIDEMIOLOGY
GILLINGS SCHOOL OF GLOBAL PUBLIC HEALTH
UNIVERSITY OF NORTH CAROLINA, CHAPEL HILL
CHAPEL HILL, NC 27599

GARFEIN, RICHARD S, MPH, PHD
PROFESSOR
DEPARTMENT OF MEDICINE
DIVISION OF GLOBAL PUBLIC HEALTH
SCHOOL OF MEDICINE
UNIVERSITY OF CALIFORNIA, SAN DIEGO
LA JOLLA, CA 92093-0507

GILL, CHRISTOPHER J, MD *
ASSOCIATE PROFESSOR
DEPARTMENT OF INTERNATIONAL HEALTH
SCHOOL OF PUBLIC HEALTH
BOSTON UNIVERSITY
BOSTON, MA 02118

GRIJALVA, CARLOS G, MD *
ASSOCIATE PROFESSOR
DEPARTMENT OF HEALTH POLICY
DIVISION OF PHARMACOEPIDEMIOLOGY
VANDERBILT UNIVERSITY SCHOOL OF MEDICINE
NASHVILLE, TN 37212

GROBMAN, WILLIAM ADAM, MD
PROFESSOR
DEPARTMENT OF OBSTETRICS AND GYNECOLOGY
NORTHWESTERN UNIVERSITY
CHICAGO, IL 60611

GUERRA, STEFANO, MD, MPH, PHD
PROFESSOR
ARIZONA RESPIRATORY CENTER
UNIVERSITY OF ARIZONA
TUCSON, AZ 85724

HARRISON, LEE H, MD
PROFESSOR
DEPARTMENTS OF MEDICINE
AND EPIDEMIOLOGY
UNIVERSITY OF PITTSBURGH
PITTSBURGH, PA 15261

HERBST-KRALOVETZ, MELISSA MARIE, PHD *
ASSOCIATE PROFESSOR
DEPARTMENT OF BASIC MEDICAL SCIENCES
UNIVERSITY OF ARIZONA CANCER CENTER
PHOENIX, AZ 85004

IVANEK MIOJEVIC, RENATA, DVM, PHD *
ASSOCIATE PROFESSOR
DEPARTMENT OF VETERINARY INTEGRATIVE BIOSCIENCES
COLLEGE OF VETERINARY MEDICINE
AND BIOMEDICAL SCIENCES
TEXAS A&M UNIVERSITY
COLLEGE STATION, TX 77843

JAIN, SONIA, PHD *
PROFESSOR
DEPARTMENT OF FAMILY AND PREVENTIVE MEDICINE
DIVISION OF BIOSTATISTICS AND BIOINFORMATICS
UNIVERSITY OF CALIFORNIA AT SAN DIEGO
LA JOLLA, CA 92093

JEFFERSON, KIMBERLY KAY, PHD *
ASSOCIATE PROFESSOR
DEPARTMENT OF MICROBIOLOGY
AND IMMUNOLOGY
VIRGINIA COMMONWEALTH UNIVERSITY
RICHMOND, VA 23298

JONES, RACHAEL MARY, PHD *
ASSISTANT PROFESSOR
DEPARTMENT OF ENVIRONMENTAL AND OCCUPATIONAL
HEALTH SCIENCES
SCHOOL OF PUBLIC HEALTH
UNIVERSITY OF CHICAGO
CHICAGO, IL 60612

KENNEDY, RICHARD B, PHD *
ASSOCIATE PROFESSOR
DIVISION OF GENERAL INTERNAL MEDICINE
MAYO CLINIC AND FOUNDATION
ROCHESTER, MN 55905

KILGORE, PAUL EVAN, MD *
ASSOCIATE PROFESSOR DIRECTOR OF RESEARCH
EUGENE APPLEBAUM COLLEGE OF PHARMACY
AND HEALTH SCIENCES
WAYNE STATE UNIVERSITY
DETROIT, MI 48201

KISSINGER, PATRICIA J, BSN, MPH, PHD
PROFESSOR
DEPARTMENT OF EPIDEMIOLOGY
SCHOOL OF PUBLIC HEALTH AND TROPICAL MEDICINE
TULANE UNIVERSITY
NEW ORLEANS, LA 70112

LANE, ROBERT H., MD *
PROFESSOR
THE BARRI L AND DAVID J DRURY CHAIR
DEPARTMENT OF PEDIATRICS
MEDICAL COLLEGE OF WISCONSIN
MILWAUKEE, WI 53201

LEVIN, ALBERT M, MPH, PHD
ASSOCIATE SCIENTIST
DEPARTMENT OF PUBLIC HEALTH SCIENCES
HENRY FORD HEALTH SYSTEM
ANN ARBOR, MI 48105

LIN, SHAO, MD, MPH, PHD
PROFESSOR
DEPARTMENT OF ENVIRONMENTAL HEALTH SCIENCE
ASSOCIATE DIRECTOR OF GLOBAL HEALTH RESEARCH
SCHOOL OF PUBLIC HEALTH
UNIVERSITY OF ALBANY, STATE UNIVERSITY OF NEW YORK
RENSSELAER, NY 12144

MARSIT, CARMEN JOSEPH, PHD
PROFESSOR
DEPARTMENT OF ENVIRONMENTAL HEALTH
ROLLINS SCHOOL OF PUBLIC HEALTH
EMORY UNIVERSITY
ATLANTA, GA 30322

NOLEN, TRACY L, DRPH *
SENIOR RESEARCH STATISTICAN
CENTER FOR CLINICAL RESEARCH NETWORK
COORDINATION
BIOSTATISTICS AND EPIDEMIOLOGY DIVISION
RTI INTERNATIONAL
RESEARCH TRIANGLE PARK, NC 27709

NOONAN, CURTIS WILLIAM, PHD
PROFESSOR
DIRECTOR, GRADUATE EDUCATION AND RESEARCH
COLLEGE OF HEALTH PROFESSIONS AND
BIOMEDICAL SCIENCES
UNIVERSITY OF MONTANA, MISSOULA
MISSOULA, MT 59812

PHIPATANAKUL, WANDA, MD *
PROFESSOR
DEPARTMENT OF IMMUNOLOGY AND ALLERGY
CHILDREN'S HOSPITAL
BOSTON, MA 02115

REICH, BRIAN J., PHD *
ASSOCIATE PROFESSOR
DEPARTMENT OF STATISTICS
NORTH CAROLINA STATE UNIVERSITY
RALEIGH, NC 27695

REMAIS, JUSTIN VICTOR, PHD *
ASSOCIATE PROFESSOR
SCHOOL OF PUBLIC HEALTH
UNIVERSITY OF CALIFORNIA
BERKLEY, CA 94720

ROHANI, PEJMAN, PHD
PROFESSOR
DEPARTMENT OF INFECTIOUS DISEASES
ODUM SCHOOL OF ECOLOGY
UNIVERSITY OF GEORGIA
ATHENS, GA 30602

SATHYANARAYANA, SHEELA, MD *
ASSOCIATE PROFESSOR
DEPARTMENT OF ENVIRONMENTAL AND
OCCUPATIONAL HEALTH SCIENCES
SCHOOL OF PUBLIC HEALTH
UNIVERSITY OF WASHINGTON
SEATTLE, WA 98121

SHARMA, SUNITA, MD *
ASSOCIATE PROFESSOR
DIV PULMONARY SCIENCES & CRITICAL CARE MEDICINE
UNIVERSITY OF COLORADO DENVER
AURORA, CO 80045-2560

SILVEYRA, PATRICIA, PHD *
ASSISTANT PROFESSOR
DEPARTMENT OF PEDIATRICS
COLLEGE OF MEDICINE
PENNSYLVANIA STATE UNIVERSITY
HERSHEY, PA 17033

SLAUGHTER, JAIME CATHERINE, PHD *
ASSISTANT PROFESSOR
COLLEGE OF NURSING AND HEALTH PROFESSIONS
DREXEL UNIVERSITY
PHILEDELPHIA, PA 19102

SMITH, AMBER M., PHD *
ASSISTANT PROFESSOR
DEPARTMENT OF PEDIATRICS
INSTITUTE FOR THE STUDY OF HOST PATHOGEN SYSTEMS
UNIVERSITY OF TENNESSEE HEALTH SCIENCE CENTER
MEMPHIS, TN 38163

STEIN, CATHERINE MARIE, PHD *
ASSOCIATE PROFESSOR
POPULATION AND QUANTITATIVE HEALTH
CENTER FOR PROTEOMICS & BIOINFORMATICS, AND
TUBERCULOSIS RESEARCH UNIT
CASE WESTERN RESERVE UNIVERSITY
CLEVELAND, OH 44106

WEI, PENG, PHD *
ASSOCIATE PROFESSOR
DEPARTMENT OF BIOSTATISTICS
THE MD ANDERSON CANCER CENTER
UNIVERSITY OF TEXAS
HOUSTON, TX 77030

WING, DEBORAH A, MBA, MD
SENIOR CLIENT PARTNER
ACADEMIC HEALTH CENTER PRACTICE
KORN FERRY INSTITUTE
ORANGE, CA 92868

MAIL REVIEWER(S)

CECIL, KIM M, PHD
PROFESSOR
DEPARTMENTS OF RADIOLOGY AND PEDIATRICS
NEUROSCIENCE AND ENVIRONMENTAL HEALTH
CINCINNATI CHILDREN'S HOSPITAL MEDICAL CENTER
CINCINNATI, OH 45229

LEDERER, DAVID J, MD, MS
ASSOCIATE PROFESSOR
DEPARTMENTS OF MEDICINE AND EPIDEMIOLOGY
COLUMBIA UNIVERSITY MEDICAL CENTER
NEW YORK, NY 10032

MAUPOME, GERARDO, DDS, PHD
PROFESSOR
RICHARD M FAIRBANKS SCHOOL OF PUBLIC HEALTH
INDIANA UNIVERSITY
INDIANAPOLIS, IN 46202-2876

SANDERS, DON B, MD
ASSOCIATE PROFESSOR OF PEDIATRICS
SECTION OF PEDIATRIC PULMONOLOGY,
ALLERGY, AND SLEEP MEDICINE
INDIANA UNIVERSITY
INDIANAPOLIS, IN 46202

ZHOU, LI, MBBS, PHD
ASSOCIATE PROFESSOR
DEPARTMENT OF MEDICINE
BRIGHAM AND WOMEN'S HOSPITAL
HARVARD MEDICAL SCHOOL
SONERVILLE, MA 02145

SCIENTIFIC REVIEW OFFICER

STEELE, LISA, PHD
SCIENTIFIC REVIEW OFFICER
CENTER FOR SCIENTIFIC REVIEW
NATIONAL INSTITUTES OF HEALTH
BETHESDA, MD 20892

EXTRAMURAL SUPPORT ASSISTANT

NAEGER, CATHERINE MLIS
EXTRAMURAL SUPPORT ASSISTANT
CENTER FOR SCIENTIFIC REVIEW
NATIONAL INSTITUTES OF HEALTH
BETHESDA, MD 20892

* Temporary Member. For grant applications, temporary members may participate in the entire meeting or may review only selected applications as needed.

Consultants are required to absent themselves from the room during the review of any application if their presence would constitute or appear to constitute a conflict of interest.
